# Supplementary material for: The effect of carbonic anhydrase on foraminiferal Mg/Ca
Source: PeerJ. 2024 Nov 29;12:e18458. doi: 10.7717/peerj.18458 (PMC11610475; doi:10.7717/peerj.18458)
Supplement: Supplemental Information 1 — Inorganic carbon chemistry, growth and calcite chemistry. [file peerj-12-18458-s001.docx]

**Supplement to De Goeyse et al.**

**The effect of carbonic anhydrase on foraminiferal Mg/Ca**

Table S1: chemical parameters of the prepared seawater (all measured, plus the calculated [CO_3_^2-^]. For each of the two treatments (‘control’ and ‘AZ added’), there were incubations at four different *p*CO_2_ concentrations (400, 800, 1200 and 1600 ppm). Shading indicates the triplicate measurements: i.e. adjacent lines with same grey shading are subsampled from the same flask.

| Treatment | *p*CO_2_ | DIC | TA | [CO_3_^2-^] calc. | PO_4_ | NH_4_ | NO_3_+NO_2_ | NO_2_ | NO_3_ |
| --- | --- | --- | --- | --- | --- | --- | --- | --- | --- |
|  | ppm | μmol/l | μeql/l | μmol/kg | μmol/l | μmol/l | μmol/l | μmol/l | μmol/l |
| Control | 400 | 1994 | 2275 | 184 |  |  |  |  |  |
|  |  | 1972 | 2265 | 191 |  |  |  |  |  |
|  |  | 1987 | 2289 | 198 |  |  |  |  |  |
|  |  | 1980 | 2286 | 200 | 0.015 | 0.53 | 0.53 | 0.420 | 0.11 |
|  |  | 1981 | 2258 | 181 |  |  |  |  |  |
|  |  | 1953 | 2238 | 185 |  |  |  |  |  |
|  |  | 1982 | 2282 | 196 | 0.017 | 0.56 | 0.54 | 0.411 | 0.13 |
|  |  | 1996 | 2267 | 178 |  |  |  |  |  |
|  |  | 1975 | 2269 | 192 |  |  |  |  |  |
|  |  | 1988 | 2294 | 200 |  |  |  |  |  |
|  |  | 1984 | - | - | 0.005 | 0.91 | 0.46 | 0.409 | 0.05 |
|  |  | 1970 | 2285 | 205 | 0.005 | 0.54 | 0.63 | 0.601 | 0.03 |
|  |  | - | 2290 | - | 0.040 | 5.13 | 0.97 | 0.480 | 0.49 |
|  | 800 | 2120 | 2263 | 103 |  |  |  |  |  |
|  |  | 2098 | 2246 | 105 |  |  |  |  |  |
|  |  | 2117 | - | - |  |  |  |  |  |
|  |  | 2124 | - | - | 0.027 | 1.86 | 0.88 | 0.603 | 0.28 |
|  |  | 2136 | 2275 | 101 |  |  |  |  |  |
|  |  | 2110 | 2275 | 115 |  |  |  |  |  |
|  |  | 2127 | 2279 | 108 | 0.007 | 0.73 | 0.40 | 0.244 | 0.16 |
|  |  | 2126 | 2271 | 104 |  |  |  |  |  |
|  |  | 2100 | 2257 | 110 |  |  |  |  |  |
|  |  | 2149 | 2299 | 108 | 0.006 | 5.68 | 0.66 | 0.593 | 0.07 |
|  |  | 2128 | 2293 | 116 | 0.005 | 0.39 | 0.09 | 0.046 | 0.05 |
|  |  | 2135 | 2298 | 115 | 0.005 | 0.37 | 0.77 | 0.621 | 0.15 |
|  | 1200 | 2171 | 2276 | 83 |  |  |  |  |  |
|  |  | 2202 | 2287 | 74 |  |  |  |  |  |
|  |  | 2188 | 2289 | 81 |  |  |  |  |  |
|  |  | 2190 | 2288 | 80 | 0.007 | 0.69 | 0.54 | 0.456 | 0.09 |
|  |  | 2179 | 2272 | 77 |  |  |  |  |  |
|  |  | 2172 | 2261 | 75 |  |  |  |  |  |
|  |  | 2185 | 2279 | 74 | 0.014 | 0.63 | 0.52 | 0.382 | 0.14 |
|  |  | 2181 | 2276 | 78 |  |  |  |  |  |
|  |  | 2195 | 2289 | 78 |  |  |  |  |  |
|  | 1600 | 2202 | 2280 | 70 |  |  |  |  |  |
|  |  | 2223 | 2263 | 53 |  |  |  |  |  |
|  |  | 2228 | 2292 | 64 |  |  |  |  |  |
|  |  | 2233 | 2286 | 59 | 0.008 | 0.69 | 0.58 | 0.419 | 0.16 |
|  |  | 2214 | 2286 | 67 |  |  |  |  |  |
|  |  | 2218 | 2273 | 60 |  |  |  |  |  |
|  |  | 2227 | 2291 | 64 | 0.034 | 1.11 | 0.51 | 0.237 | 0.27 |
|  |  | 2198 | 2268 | 66 |  |  |  |  |  |
|  |  | 2217 | 2263 | 56 |  |  |  |  |  |
| AZ added | 400 | 1956 | 2309 | 230 | 0.012 | 0.08 | 0.11 | 0.067 | 0.05 |
|  |  | 1996 | 2293 | 195 | 0.136 | 1.80 | 1.20 | 0.712 | 0.49 |
|  |  | 1984 | 2295 | 203 |  |  |  |  |  |
|  |  | 1976 | 2302 | 213 | 0.005 | 0.07 | 0.09 | 0.015 | 0.07 |
|  |  | 1992 | 2328 | 220 | 0.005 | 0.21 | 0.26 | 0.181 | 0.08 |
|  |  | 1974 | 2314 | 222 | 0.007 | 0.47 | 0.98 | 0.539 | 0.44 |
|  |  | 1992 | 2315 | 212 | 0.080 | 0.34 | 0.55 | 0.025 | 0.52 |
|  |  | 1978 | 2303 | 212 | 0.005 | 0.08 | 0.06 | 0.020 | 0.04 |
|  |  | 1981 | 2308 | 214 | 0.023 | 0.17 | 0.15 | 0.048 | 0.10 |
|  | 800 | - | 2321 | - | 0.026 | 0.42 | 0.65 | 0.603 | 0.04 |
|  |  | 2130 | 2287 | 111 | 0.109 | 0.26 | 0.71 | 0.286 | 0.43 |
|  |  | 2131 | 2299 | 118 |  |  |  |  |  |
|  |  | 2134 | 2307 | 121 | 0.005 | 0.42 | 0.20 | 0.165 | 0.04 |
|  |  | 2137 | 2312 | 122 | 0.005 | 0.11 | 1.18 | 0.809 | 0.37 |
|  |  | 2141 | 2303 | 114 | 0.008 | 1.85 | 0.25 | 0.140 | 0.11 |
|  |  | 2141 | 2322 | 126 | 0.008 | 2.01 | 1.25 | 0.661 | 0.59 |
|  |  | 2146 | 2321 | 122 | 0.005 | 0.40 | 0.40 | 0.323 | 0.08 |
|  |  | 2135 | 2301 | 117 | 0.005 | 0.01 | 0.16 | 0.114 | 0.05 |
|  | 1200 | 2147 | 2298 | 108 | 0.014 | 1.21 | 0.68 | 0.663 | 0.02 |
|  |  | 2188 | 2282 | 78 | 0.119 | 0.23 | 0.73 | 0.217 | 0.51 |
|  |  | 2188 | 2290 | 82 |  |  |  |  |  |
|  |  | 2209 | 2318 | 86 | 0.005 | 0.04 | 1.38 | 1.127 | 0.25 |
|  |  | 2199 | 2309 | 86 | 0.005 | 0.08 | 0.45 | 0.413 | 0.03 |
|  |  | 2190 | 2305 | 89 | 0.007 | 0.23 | 1.16 | 0.906 | 0.25 |
|  |  | 2209 | 2316 | 85 | 0.005 | 1.77 | 1.84 | 1.241 | 0.60 |
|  |  | 2209 | 2306 | 80 | 0.005 | 0.25 | 0.84 | 0.318 | 0.52 |
|  |  | 2210 | 2310 | 81 | 0.005 | 0.36 | 1.08 | 1.012 | 0.07 |
|  | 1600 | 2244 | 2299 | 60 | 0.025 | 0.53 | 1.04 | 0.531 | 0.50 |
|  |  | 2242 | 2294 | 59 |  |  |  |  |  |
|  |  | 2240 | 2300 | 62 |  |  |  |  |  |
|  |  | 2199 | 2320 | 92 | 0.050 | 1.21 | 0.20 | 0.149 | 0.05 |
|  |  | 2239 | 2298 | 62 | 0.133 | 0.39 | 0.28 | 0.064 | 0.21 |
|  |  | 2268 | 2324 | 61 | 0.012 | 0.06 | 0.60 | 0.527 | 0.07 |
|  |  | 2261 | 2312 | 59 | 0.005 | 0.04 | 0.13 | 0.104 | 0.03 |
|  |  | 2241 | 2332 | 78 | 0.054 | 0.07 | 0.61 | 0.501 | 0.11 |
|  |  | 2253 | 2310 | 61 | 0.005 | 1.32 | 0.23 | 0.028 | 0.20 |
|  |  | 2254 | 2308 | 60 | 0.005 | 0.49 | 0.61 | 0.520 | 0.09 |
|  |  | 2248 | 2298 | 58 | 0.005 | 0.19 | 0.90 | 0.561 | 0.34 |

Table S2: comparison between Na/Ca, Mg/Ca and Sr/Ca between solution-ICP-MS and LA-ICP-MS. Data per treatment (i.e. control or AZ added) was combined prior to statistical testing.

|  | Control | | | AZ added | | |
| --- | --- | --- | --- | --- | --- | --- |
|  | Average (mmol/mol) | SD | n | Average (mmol/mol) | SD | n |
| Solution-ICP-MS | | | | | | |
| Na/Ca | 8.47 | 0.89 | 14 | 13.3 | 3.0 | 4 |
| Mg/Ca | 25.2 | 3.6 | 14 | 79.1 | 6.6 | 4 |
| Sr/Ca | 1.63 | 0.076 | 14 | 1.77 | 0.051 | 4 |
| LA-ICP-MS | | | | | | |
| Na/Ca | 7.84 | 2.3 | 360 | 13.5 | 5.2 | 37 |
| Mg/Ca | 30.2 | 8.7 | 360 | 94.8 | 12 | 38 |
| Sr/Ca | 1.58 | 0.14 | 360 | 1.67 | 0.086 | 38 |

Table S3: results of a t-Test, assuming equal variances, alpha = 0.05.

|  | Control |  |  |
| --- | --- | --- | --- |
|  | Na/Ca | Mg/Ca | Sr/Ca |
| df | 372 | 372 | 372 |
| P-value | 0.14 | 0.034 | 0.21 |
| T stat | -1.44 | 2.13 | -1.26 |
|  | Az added |  |  |
|  | Na/Ca | Mg/Ca | Sr/Ca |
| df | 39 | 40 | 40 |
| P-value | 0.93 | 0.018 | 0.039 |
| T stat | 0.0849 | 2.46 | -2.13 |
